# Using multiplexed regulation of luciferase activity and GFP translocation to screen for FOXO modulators

**DOI:** 10.1186/1471-2121-10-14

**Published:** 2009-02-25

**Authors:** Fabian Zanella, Aranzazú Rosado, Beatriz Garcia, Amancio Carnero, Wolfgang Link

**Affiliations:** 1 Experimental Therapeutics Programme, Centro Nacional de Investigaciones Oncologicas (CNIO), Melchor Fernandez Almagro 3, 28029 Madrid, Spain; 2 Current address: NKI-AVL, Plesmanlaan 121, 1066 CX, Amsterdam, The Netherlands

## Abstract

**Background:**

Independent luciferase reporter assays and fluorescent translocation assays have been successfully used in drug discovery for several molecular targets. We developed U2transLUC, an assay system in which luciferase and fluorescent read-outs can be multiplexed to provide a powerful cell-based high content screening method.

**Results:**

The U2transLUC system is based on a stable cell line expressing a GFP-tagged FOXO transcription factor and a luciferase reporter gene under the control of human FOXO-responsive enhancers. The U2transLUC assay measures nuclear-cytoplasmic FOXO shuttling and FOXO-driven transcription, providing a means to analyze these two key features of FOXO regulation in the same experiment. We challenged the U2transLUC system with chemical probes with known biological activities and we were able to identify compounds with translocation and/or transactivation capacity.

**Conclusion:**

Combining different biological read-outs in a single cell line offers significant advantages over conventional cell-based assays. The U2transLUC assay facilitates the maintenance and monitoring of homogeneous FOXO transcription factor expression and allows the reporter gene activity measured to be normalized with respect to cell viability. U2transLUC is suitable for high throughput screening and can identify small molecules that interfere with FOXO signaling at different levels.

## Background

Forkhead box O (FOXO) proteins are emerging as transcriptional integrators of pathways that regulate a variety of cellular processes, including differentiation, metabolism, stress response, cell cycle and apoptosis [1-3]. FOXO transcription factors have been proposed to act as *bona fide *tumor suppressors due to their inhibitory effects on cell cycle and survival [4], properties mediated by their binding as monomers to consensus DNA binding sites. Their transcriptional activity is governed by a network of signaling events, the best recognized of which is the phosphorylation of FOXO proteins at three highly conserved serine and threonine residues by Akt that provokes its association with 14-3-3 protein and in turn, the nuclear exclusion of phospho-FOXO. However, the relocation of FOXO from the nucleus to the cytoplasm alone cannot account for the inhibitory effect of PI3K/Akt signaling on FOXO activity since a nuclear form of FOXO1 in which the nuclear export sequence is disrupted is still inhibited by the PI3K/Akt pathway [5]. Indeed, the introduction of a negative charge in the positively charged DNA binding domain by means of FOXO phosphorylation at the second of the three Akt consensus sites inhibits DNA binding of FOXO [6,7]. The FOXO DNA interaction is also regulated by the transfer of acetyl groups to lysine residues in FOXO proteins by the histone acetyltransferases (HATs) CBP and p300 [2], which alters the DNA binding capacity of FOXO1 and FOXO3a [8]. Conversely, Sirt1 deacetylases deacetylate FOXO factors and regulate their DNA binding at specific target genes. Taken together, these observations suggest that translocation and transactivation are different and separate means to regulate FOXO. However, large scale tools are not available to assess the different levels of FOXO regulation. Therefore systematic chemical genetic or loss of function studies to investigate the complex regulation of FOXO factors have been limited only to certain aspects [9].

In anticancer drug discovery, much effort is directed towards identifying small molecule inhibitors of PI3K/Akt signaling using cell based high content screening. In particular, monitoring the intracellular localization of FOXO transcription factors has been used to screen large numbers of small molecules [10,11]. Despite being commonly used as a reporter-gene system in drug discovery, luciferase-based transcriptional assays have not been applied to massive compound screens for PI3K/Akt inhibitors. Inhibiting the PI3K/Akt pathway causes FOXO3a to remain in the cell nucleus and subsequently, it induces the transcription of downstream genes. To take advantage of these regulatory features we generated the stable U2transLUC dual assay cell line that expresses FOXO responsive luciferase activity and GFP labelled FOXO. Thus, U2transLUC can be used to simultaneously monitor the intracellular translocation and the transcriptional activity of FOXO proteins. We have used this cell line in an attempt to identify small molecules that interfere with FOXO signaling.

## Results

### Generation and testing of luciferase reporter gene constructs

**Figure 1:**
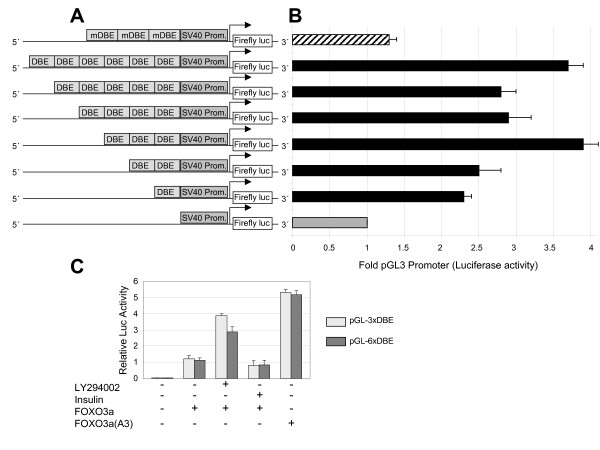
**FOXO-driven transcriptional activity measured by Luciferase production from different reporter gene constructs**. **A**. Schematic diagram of the luciferase reporter constructs used in this study. One to six copies of the DBE binding cassettes were cloned upstream of the luciferase reporter gene in the pGL3-Promoter vector to create the plasmids pGL-1xDBE, pGL-2xDBE, pGL-3xDBE, pGL-4xDBE, pGL-5xDBE and pGL-6xDBE. The empty pGL3-Promoter vector and a reporter plasmid that carries three copies of a mutated DBE (mDBE) region were used as control plasmids. B. Each construct was transiently co-transfected with plasmids encoding FOXO3a and Renilla luciferase into U2OS cells, and the luciferase activities were determined as described in the "Methods" section. The data were normalized to the Renilla luciferase (phRG-TK vector) reporter construct and expressed relative to the normalized activity of the pGL3-Promoter vector. The results are given as the mean ± SEM of three independent experiments performed in triplicate. C. Basal and induced FOXO-dependent transcriptional activity conferred by three or six copies of the DBE binding cassette (grey and black bars, respectively). The plasmids pGL-3xDBE or pGL-6xDBE were transiently co-transfected into U2OS cells with plasmids encoding wild type FOXO3a or the constitutively active FOXO3a-A3 and Renilla luciferase. Where indicated cells were treated with 20 μM LY294002 or insulin for 6 hours.

FOXO proteins drive the transcription of downstream genes by binding to the TTGTTTAC FOXO responsive enhancer element, generally referred to as a daf-16 family protein-binding element (DBE) [12]. To take advantage of these regulatory features, we engineered several luciferase reporter constructs that contained one to six copies of the DBE consensus cassette in front of a SV40 minimal viral promoter that was linked to a luciferase reporter gene. The resulting reporter gene construct were designated as pGL-1xDBE, pGL-2xDBE, pGL-3xDBE, pGL-4xDBE, pGL-5xDBE and pGL-6xDBE (Fig. 1A), and the luciferase activity driven by FOXO from these constructs was evaluated after they were transiently transfected into U2OS osteosarcoma cells. Since endogenous FOXO3a is only weakly expressed in these cells, ectopic FOXO3a also had to be expressed to achieve acceptable basal levels of luciferase activity (data not shown). In transient co-transfection assays, all the luciferase reporter constructs that carried FOXO responsive DBE elements produced a significant increase in luciferase activity when compared to the empty pGL3-Promoter vector. Constructs that contained three or six copies of the DBE element conferred significantly stronger FOXO-dependent transcriptional activity than those with one, two, four or five copies (Fig. 1B). A reporter plasmid that carried three copies of a mutated DBE (pGL-3xDBEmut) element did not promote significant luciferase activity, confirming the specificity of the original constructs for FOXO-mediated transcriptional activity.

In order to evaluate the responsiveness of the reporter constructs to the inhibition of the PI3K/Akt pathway, we transiently co-transfected the pGL-3xDBE or pGL-6xDBE construct with FOXO3a into U2OS cells and then treated them with the PI3K inhibitor, LY294002 (20 μM). Inhibition of the PI3K pathway increased FOXO-dependent transcription from both the pGL-3xDBE and pGL-6xDBE constructs approximately 3-fold (Figure 1B). By contrast, activation of the PI3K/Akt signaling pathway following exposure to insulin decreased this luciferase activity. However, the differences between the transcriptional activity of FOXO3a in untreated and insulin treated U2OS cells were small, indicating that steady-state level of PI3K/Akt activity was already quite high. In addition, to confirm the specificity of our system we examined the effect of constitutive FOXO activation by co-expressing the pGL-3xDBE or pGL-6xDBE reporter construct with FOXO3a-A3, a constitutively active form of FOXO3a in which the three PI3K-dependent phosphorylation sites have been mutated to alanine. The expression of FOXO3a-A3 induced a strong increase in pGL-3xDBE or pGL-6xDBE driven luciferase activity. Together, these data indicate that the firefly luciferase-based read-out of FOXO activity is very specific, and that it provides a large window to measure any upregulation in the response (e.g. upon the inhibition of the PI3K/Akt pathway). Since the responsiveness of the triple tandem repeat of DBE (pGL-3xDBE) to PI3K inhibition was slightly higher than that of pGL-6xDBE, the p3xDBE-luc construct was used to generate the stable cell assay line, U2transLUC.

### Generation of the assay cell line, U2transLUC

**Figure 2:**
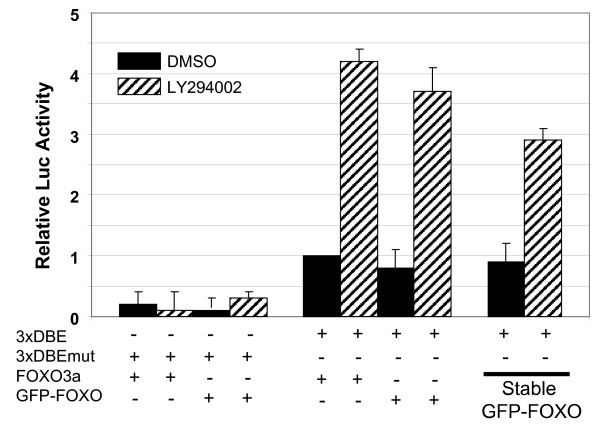
**FOXO3a and GFP-FOXO trigger a similar induction of luciferase activities**. pGL-3xDBE or pGL-3xDBEmut were transiently co-transfected with plasmids encoding FOXO3a or GFP-FOXO3a into U2OS cells. U2foxRELOC cells that stably express the GFP-FOXO3a protein (9) were transiently transfected with pGL-3xDBE. LY294002 (20 μM, hatched bars) or DMSO (black bars) were added to the transfected cells six hours before processing the cells to assay the luciferase assay. The values are the means ± SEM of relative luciferase activities from three independent experiments performed in triplicates.

We recently reported the use of a high-throughput cellular imaging assay to monitor the nucleo-cytoplasmic translocation of a GFP-FOXO3a fusion protein in U2OS cells (U2foxRELOC) for a chemical genetic study [9]. In order to generate an assay system that combines the benefits of large-scale image-based analysis and luciferase-based end point measurements, we examined the compatibility of transcriptional and translocational reporters expressed in a single cell line. We first explored whether the GFP-tagged FOXO protein is still capable of driving luciferase expression from FOXO responsive reporters. We compared the reporter activity of the pGL-3xDBE construct or the mutated version (pGL-3xDBEmut) co-expressed with either untagged FOXO3a wt protein or EGFP-tagged FOXO3a. Hence, we transiently transfected untagged FOXO3a and EGFP-FOXO3a into U2OS cells and measured the luciferase activities 4 hours after exposure to LY294002 or DMSO. The N-terminal EGFP tag did not significantly affect transcriptional function of FOXO3a in U2OS cells (Fig. 2) indicating the compatibility of luminescent and fluorescent read-outs in a single cell line. In addition, we compared the level of luciferase activity driven by the pGL-3xDBE construct in the context of stable EGFP-FOXO3a expression in U2foxRELOC cells [9] and with the transient co-expression of the fluorescent fusion protein. The luciferase expression in the presence of stable EGFP-FOXO3a expression was only slightly lower than that in U2OS cells transiently overexpressing EGFP-FOXO3a. To stably integrate the pGL-3xDBE construct into the genome of U2foxRELOC cells, we modified the pGL3-promoter construct by inserting a puromycin-resistance cassette to obtain pGLpuro-3xDBE. Puromycin-resistant stable clones were generated (U2transLUC) and one was chosen for further use based on FOXO-induced luciferase activity.

### The use of GFP fusion protein for normalization

**Figure 3:**
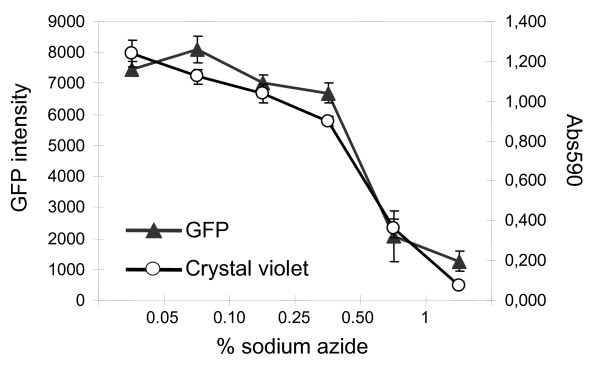
**Viability of U2transLUC cells using crystal violet assay or by monitoring the GFP-intensity per well**. U2transLUC cells were exposed to 0.05%, 0.10%, 0.25%, 0.50% or 1% sodium azide for three hours and processed as described in the "Methods" section. The values are the means (± SD) of GFP intensity per well or the absorbance at 595 nm from three independent experiments.

We examined whether the expression of the GFP fusion protein could be used to normalize the transcriptional reporter gene to the changes in cell viability. U2transLUC cells were seeded and allowed to attach to the wells overnight. Subsequently, they were incubated with increasing doses of sodium azide, a known inhibitor of the respiratory chain that provokes necrosis in many cell types [13,14]. Cell viability was estimated through a crystal violet assay or through the intensity of the fluorescence measured in each well. Both methods take advantage of the fact that when epithelial cells undergo apoptosis or necrosis, they detach from plates and are no longer detectable by these assays. After a 6 hour exposure to 0.05% sodium azide the viability of U2transLUC cells was slightly reduced with no significant differences between crystal violet or fluorescent intensity read-outs. Higher doses of sodium azide dramatically compromised U2transLUC viability, as reflected by both measures of viability. A normalization procedure using crystal violet staining or the GFP-FOXO mediated fluorescent signal resulted in similar indices of viability (Fig. 3), indicating that the fluorescent reporter protein can be used to monitor the viability of U2transLUC cells.

### Multiplexed U2transLUC assay

**Figure 4:**
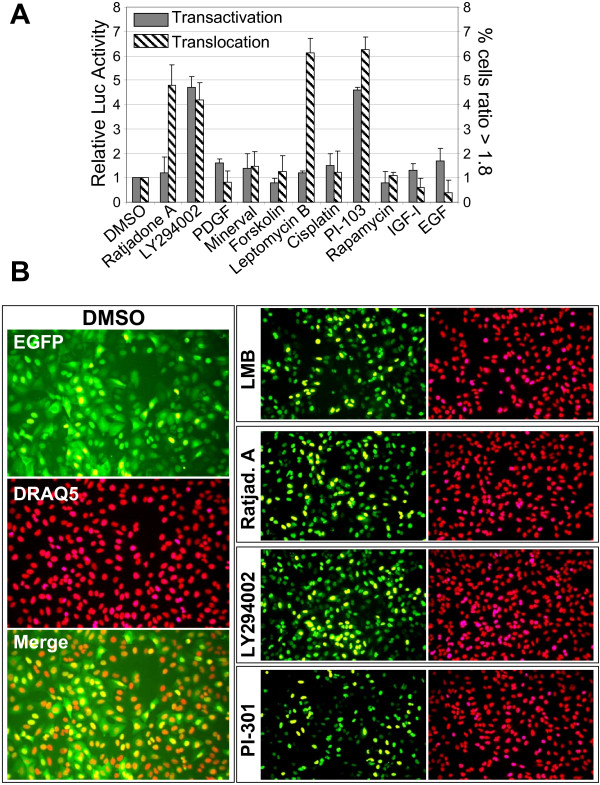
**Validation of U2transLUC assay using a panel of test compounds**. **A**. The nuclear accumulation of the GFP-FOXO reporter protein and FOXO driven luciferase activity induced by the test compounds. We exposed U2transLUC cells to three different concentrations of the test compounds. The results shown here are the values obtained from the treatment of U2transLUC cells with 4 nM Ratjadone A, 20 μM LY294002, 1 ng/ml PDGF, 100 μM Minerval, 1 μM Forskolin, 4 nM Leptomycin B, 30 μM Cisplatin, 100 nM PI-103, 0.5 nM Rapamycin, 20 ng/ml IGF, 5 ng/ml EGF and Dimethyl sulfoxide (DMSO) as a negative control. The grey bar graphs and the corresponding left hand y-axis depict the values in relation to the control values normalized to the corresponding measure of viability values. The relative luciferase activity was calculated dividing the value obtained for Firefly luciferase activity for each well by the average GFP intensity from the same well. The right hand y-axis and the hatched bars indicate the percentage of cells in each well exhibiting nuclear/cytoplasmic (Nuc/Cyt) ratios of fluorescence intensity greater than 1.8 normalized to the percentage in DMSO-treated wells. **B**. Representative images of treated cells using high throughput format of the U2transLUC system. Images of cells expressing GFP-FOXO and stained with DRAQ5 were obtained by automated microscopy 2 hour after drug exposure. The images correspond to U2transLUC cells exposed to 4 nM Ratjadone A, 4 nM Leptomycin B, 20 μM LY294002, 100 nM PI-103 and DMSO. The images are shown from the GFP and DRAQ5 channels, as well as the corresponding merged image in the case of the DMSO treated control cells.

The U2transLUC-based assay was formatted for 96-well plates and the workflow has been automated. To assess the specificity of the transcriptional and the translocational read-out, we challenged the U2transLUC system with small molecule inhibitors under living cell conditions in order to observe the translocation of the fluorescent reporter protein and luciferase activity as the transcriptional end point. We exposed U2transLUC cells to a panel of test compounds with known biological activity. Three different doses were used that resulted in a range of final concentrations greater than two orders of magnitude around the IC_50 _value for each individual compound. The live cells were stained with the fluorescent nuclear dye DRAQ5 and images were obtained on an automatic microscope. The nuclear/cytoplasmic (Nuc/Cyt) fluorescence intensity ratios were determined and the percentage of cells per well displaying nuclear translocation or the inhibition of nuclear export was calculated. Compounds that induced the nuclear accumulation of the fluorescent signal above 60% of that obtained from wells treated with LY294002 were considered as hits. Several compounds known to inhibit PI3K activity or the nuclear export machinery fulfilled these criteria (Fig 4A). As expected from the results of a previous study in U2foxRELOC cells [9,15], the PI3K inhibitors PI-103 and LY294002 were capable of inducing FOXO translocation into the nucleus of U2transLUC cells. By contrast, known activators of the PI3K/Akt pathway produced little nuclear localization of GFP-FOXO, including EGF, IGF and PDGF. The exposure of U2transLUC cells to the nuclear export inhibitors leptomycin B or ratjadone A provoked the nuclear accumulation of fluorescent signal, as shown previously for U2foxRELOC cells [9,15]. Conversely, through the measurement of the FOXO-dependent production of firefly luciferase, leptomycin B and ratjadone A exerted no significant effect on the transcriptional activity of FOXO (Fig 4B). Finally, exposure to PI-103 and LY294002 triggered a dramatic increase in FOXO-dependent luciferase activity. Together, these data show that the U2transLUC assay serves as an automated high throughput assay that can identify inhibitors of PI3K/Akt signaling through the high content analysis of protein translocation in conjunction with an independent analysis of the transcriptional activity of FOXO transcriptions factors.

## Discussion and conclusion

FOXO transcription factors are tightly regulated at different levels, including their intracellular translocation and transcriptional activity [1,3]. Here we report the generation of a multiplexed assay system that is capable of simultaneously monitoring these signaling events. The assay system is based on U2transLUC cells that stably express two different reporter constructs. The intracellular translocation of FOXO factors is followed using a fluorescent tagged FOXO reporter protein, whereas transcriptional activity is monitored via FOXO-dependent luciferase production. We show here that the fluorescent and luminescent read-outs are compatible in the same cell.

The design of the U2transLUC screen presented here offers some major advantages over more classical cellular assays. Primary and secondary screens of small molecule compounds are usually performed in different cell systems. By contrast, the U2transLUC provides the possibility of using two different read-outs within the same experiment, enabling a direct comparison of the hits from each of these. These hits might be divided into translocation, transactivation and dual hits, and they may in turn be analyzed according to a corresponding hit ranking. The U2transLUC assay does not need the introduction of additional plasmids and hence, it avoids the limitations associated with transfection procedures.

The use of the GFP-FOXO fusion protein provides a multifunctional tool that allows for versatile assay read outs, measurement of cell viability/number and sorting and maintaining a homogeneous cell assay population. We show that the fluorescent signal from the GFP-FOXO fusion protein is suitable to monitor viability and it can be used to normalize the values obtained by measuring firefly luciferase activity. Furthermore, the fluorescent FOXO functions as a reporter protein for FOXO translocation and as an effector protein that drives DBE-dependent luiferase production. On the other hand the GFP-FOXO fusion protein offers the possibility to control the level of expression of the transcriptional effector in the luciferase assay, and at the same time it serves as a sorting tool to maintain a homogenous cell population. Thus, the multifunctional use of GFP-FOXO allows also to reduce the variability of the assay. The U2transLUC is an image based high throughput assay that enables compounds that produce artifacts and cytotoxicity to be identified on a single cell basis. Finally, the U2transLUC assay design might be adaptable to any transcription factor that undergoes nucleo-cytoplasmic shuttling. We challenged the U2transLUC system with chemical agents with known biological activity and were able to identify known PI3K inhibitors as double-hits that score in the translocation and the transactivation read-out. By contrast, chemical probes that inhibit the general nuclear export machinery failed to produce an increase in luciferase activity although GFP-FOXO was trapped in the cell nucleus.

In summary, our data demonstrate that U2transLUC is a sensitive and robust assay to identify small-molecule inhibitors of signaling events that regulate the subcellular localization and/or the transcriptional activity of FOXO proteins. The signaling events identified here include PI3K/Akt signaling and nuclear export. Our data raise the expectation that a more extensive chemical interrogation of the U2transLUC read-outs could lead to the identification of new molecular targets and small molecules that might contribute to the development of more potent therapeutic agents to treat tumors.

## Methods

### Compound supply and recombinant proteins

All chemicals were purchased from commercial sources except for the PI3K inhibitor PI-103, which was synthesized following published patent specifications. Cisplatin was provided by C. Navarro, Minerval was generously provided by P. Escriba, and all other chemicals were purchased from commercial sources: LY294002, Ratjadone A, were purchased from Calbiochem (San Diego, CA); Forskolin, Leptomycin B and Rapamycin, were purchased from LC Laboratories (Woburn, MA, U.S.A.); DMSO was purchased from Sigma-Aldrich (St. Louis, USA); Epidermal growth factor (EGF), platelet-derived growth factor (PDGF) were purchased from RELIATech A.S. (Braunschweig, Germany); and human Insulin-Like Growth Factor-I (IGF-I) and human insulin were purchased from (Roche Diagnostics, Mannheim, Germany). Stock solutions of the test compounds were deposited in three different concentrations in 96-well master plates, transferred to multiple replica plates and frozen at -80°C.

### Plasmids

The luciferase reporter constructs pGL-1xDBE, pGL-2xDBE, pGL-3xDBE, pGL-4xDBE, pGL-5xDBE and pGL-6xDBE were generated by inserting one to six copies of the DBE consensus sequence [12] in front of a SV40 minimal viral promoter of the pGL3-Promoter vector (Promega). The annealed and phosphorylated oligonucleotides 5'-CTAGAAGTAAACAA-3' (1xDBE-forward) and 5'-GATCTT-GTTTAC-3' (1xDBE-reverse) or 5'-CTAGAAGTAAACAACTATGTAAACAA-3' (2xDBE-forward) and 5'-GATCTTGTTTACATAGTTGTTTACTT-3' (2xDBE-reverse) were ligated as single copy or concatemerized into NheI and BglII digested pGL3 promoter vector. In order to generate the negative control plasmid pGL-3xDBEmut, three copies of a DBE sequence that contains a point mutation that prevents FOXO binding (annealed and phosphorylated oligonucleotides (3xmDBE-forward) 5'-CTAGAAGTA-AGCAACTATGTAAG-CAACTATGTAAGCAA-3' and (3xmDBE-reverse) 5'-GAT-CTTGCTTACATAGTTGCTTACATAGTTGCT-TACATAG-3') were inserted into pGL3-Promoter vector. The constitutively active construct FOXO3a-A3, in which three PI3K-dependent phosphorylation sites have been mutated to alanine was kindly provided by Dr. M. Hu (University of Texas M. D. Anderson Cancer Center, Houston). In order to generate stable cell lines for the assay, we inserted a puromycin-resistance cassette into the pGL-3xDBE construct. This was achieved by PCR amplifying the puromycin resistance gene from pBABEpuro vector and cloning it into the SalI and BamHI sites of the pGL3-Promoter vector derived pGL-3xDBE construct, thereby obtaining pGLpuro-3xDBE.

### Cell culture

U2-OS cells obtained from the ATCC were cultivated in Dulbecco's modified Eagle's medium (DMEM), supplemented with 10% fetal bovine serum (FBS, Sigma), antibiotics and antimycotics in a humidified incubator at 37°C with 5% CO_2_. U2foxRELOC cells have been described previously [9].

### Transfection and Luciferase assays

U2OS cells were cultured in a 96-well plate (100 μl final volume per well) and transfected at 70% confluence with the plasmids indicated using the effectene transfection reagent (Qiagen). LY294002 or insulin were added individually to wells 42 hours later, at a final concentration of 20 μM or 5 mg/ml, respectively, and the cells were incubated for an additional 6 h. Luciferase assays were carried out using the Dual-Luciferase Reporter Assay System (Promega), according the manufacturer's instructions on a multilabel plate reader (Wallac Victor, Perkin-Elmer), and the ratio of *firefly*- to *Renilla*-luciferase activities was calculated. All values were presented as means ± SEM. The unpaired t-test (two-tailed) was performed for statistical analysis using the GraphPad PRISM^® ^Version 4.0 program. Differences with a p value < 0.05 were considered statistically significant.

### Generation and maintenance of U2transLUC cells

U2foxRELOC cells were transfected at 75% confluence with pGLpuro-3xDBE using the effectene transfection reagent (Qiagen). Cells were selected with puromycin (Calbiochem) for five days and the resistant colonies that best expressed the reporter constructs were then recovered and cultured, as was the most homogeneous population. Fluorescence-activated cell sorting (FACS) of GFP-FOXO expressing cells was performed on a FACSAria (BD Biosciences, San Jose, CA, USA). U2transLUC cell clones were maintained in DMEM, supplemented with 10% FBS (Sigma), antibiotics and antimycotics, 0.1 mg/ml Neomycin and 1 μg/ml puromycin. Cell cultures were maintained in a humidified incubator at 37°C with 5% CO_2_, and they were passaged when confluent using trypsin/EDTA.

### Crystal violet assay

Triplicate samples of 10^4 ^cells were seeded in 2.5 cm dishes and allowed to attach. After 24 hours, the medium was removed and replaced with culture medium with sodium azide at the concentrations indicated, while the controls remained untreated. After the appropriate time period the cells were fixed with 0.5% glutaraldehyde and stained with 1% crystal violet. After extensive washing, crystal violet was resolubilized in 10% acetic acid and quantified at 595 nm as a relative measure of cell number.

### Viability assay measuring fluorescent intensity

Samples of 10^4 ^cells per well were allowed to attach overnight in 96-well Greiner plates and the following day the culture medium was replaced with fresh medium containing the concentrations of sodium azide indicated. After a three hour treatment, the cells were washed with PBS and the fluorescent intensity was measured in a multilabel plate reader (Wallac Victor 2, Perkin-Elmer) using UV light as the excitation source, as well as a F485 CW lamp Filter and a F535 CW emission filter for 0.5 seconds per well. The values are the averages obtained from experiments carried out in triplicate.

### U2transLUC assay

U2transLUC cells were seeded at a density of 1.0 × 10^5 ^cells/ml in black-wall clear-bottom 96-well microplates (BD Biosciences) using a Titan Multidrop 384 automatic dispenser (Titertek Instruments, Inc., Huntsville, AL). The final volume of the cell suspension was 200 μl in each well. After incubation at 37°C in 5% CO_2 _for 12 hours, 2 μl of each test compound was transferred from the master plate to the assay plate. Cells were incubated in the presence of the compounds for 1 hour and the far-red fluorescent cell-permeable DNA probe, DRAQ5™ (Biostatus Ltd, Leicestershire, UK), was then added to all wells at a final concentration of 5 mM 15 minutes prior to obtaining the images. The images were acquired as described previously [16] using a BD Pathway™ 855 Bioimager equipped with a incubation chamber that provided a constant temperature of 37°C in 5% CO_2_. Images were acquired in the GFP and DRAQ5 channels using 488/10 nm EGFP excitation filter, a 515LP nm EGFP emission filter and 635/20 nm/695/55 nm DRAQ5 excitation/emission filter with a 10× dry objective. The plates were exposed for 0.066 ms (Gain 31) to acquire DAPI images and 0.55 ms (Gain 30) for GFP images. Image and data analysis was performed as described previously [15]. After image acquisition, the plates were incubated for another 5 hours at 37°C and then the average GFP intensity per well was measured as described above. Finally, U2transLUC cells were processed to measure the firefly luciferase activity using the Luciferase Assay System (Promega) on a multilabel plate reader (Wallac Victor, Perkin-Elmer) according to the manufacturer's instructions. The relative luciferase activity, as a measurement of FOXO3a's transcriptional activity was calculated dividing the value obtained for Firefly luciferase activity for each well by the the average GFP intensity from the same well.

## Authors' contributions

WL designed the research, interpreted the results and wrote the paper; AR and FZ performed the research; BG provided technical support. AC co-developed and co-refined the research. All authors read and approved the final manuscript.
